# Total Knee Revision Using the Midvastus Approach: A Case Report

**DOI:** 10.7759/cureus.83945

**Published:** 2025-05-12

**Authors:** Rayan Jamal, Hatem A Shaqroon

**Affiliations:** 1 Department of Orthopedic Surgery, Prince Mohammed Bin Abdulaziz Hospital, Ministry of National Guard Health Affairs, Medina, SAU; 2 Department of Orthopedic Surgery, King Fahad Hospital, Ministry of Health, Medina, SAU

**Keywords:** component loosening, knee osteoarthritis, midvastus approach, revision tka, total knee replacement

## Abstract

Total knee arthroplasty (TKA) is a common procedure for managing severe knee osteoarthritis. However, component loosening continues to be a common issue, frequently requiring revision surgery. Although the medial parapatellar (MPP) approach is commonly used due to the broad access it provides, it may disrupt the extensor mechanism and reduce blood flow to the patella. We present a case of a 62-year-old woman who required revision TKA (RTKA) on the left knee due to tibial component loosening seven years after her initial surgery, which had been performed via the MPP approach. We have used the midvastus (MV) approach to preserve the quadriceps tendon. This case highlights the MV approach as a viable alternative in RTKA, offering adequate exposure while minimizing soft tissue damage.

## Introduction

Knee osteoarthritis (OA) is a leading cause of chronic pain, disability, and high healthcare costs among older adults. In recent years, overweight and obese individuals are up to four times more likely to develop knee OA due to an increase in mechanical load on the bearing joint [1]. Total knee arthroplasty (TKA) utilizes an artificial prosthesis to replace a severely injured knee that has lost its normal function, eliminating pain, restoring knee stability, and improving quality of life [2].

The choice of the surgical approach in TKA could play a key role in knee prosthetic component alignment and the patient’s recovery, especially during the early postoperative period [3,4]. The medial parapatellar (MPP) approach was long regarded as the standard due to the excellent exposure it provides. However, this approach disrupts the quadriceps muscle and patellar blood supply, which may increase the risk of surgical complications such as button loosening, patellar dislocation, and anterior knee pain [5,6].

As an alternative, the midvastus (MV) approach became popular as it is associated with increased stability of the patellofemoral joint and decreased scarring of the quadriceps tendon [7]. This approach minimizes damage to peripheral blood vessels and muscles in the knee while providing a good surgical field of view [8].

A significant number of patients require revision TKA (RTKA), a procedure that replaces the previously implanted artificial knee joint or prosthesis with a new one, due to reasons such as mechanical wear (polyethylene or metal), implant loosening or breakage, malalignment, infection, instability, periprosthetic fracture, and persistent stiffness [9,10]. Studies report that the revision rate for TKA averages 12% over a 10-year period, with a significant number of patients requiring revision due to factors such as mechanical wear, aseptic loosening, infection, and instability [10]. However, the overall outcome of RTKA is generally less satisfactory than that of primary TKA because of uncertainties in success rates and multiple risk factors for failure. It is considered a demanding procedure that requires adequate exposure, implant extraction and restoration, and correction of bone loss, joint stability, and soft tissue insufficiency to achieve stable and durable reconstruction [11,12].

Surgical exposure for RTKA can be extensive, often involving incisions of at least 15 cm and sometimes exceeding 25 cm. The associated exposure and damage to the quadriceps muscle can be significant, and eversion of the patella can be difficult [13]. This case report aims to present a successful revision of TKA using the MV approach in a patient with tibial component loosening, highlighting its advantages over the traditional MPP approach in terms of surgical exposure and postoperative outcomes.

## Case presentation

**Figure 1:**
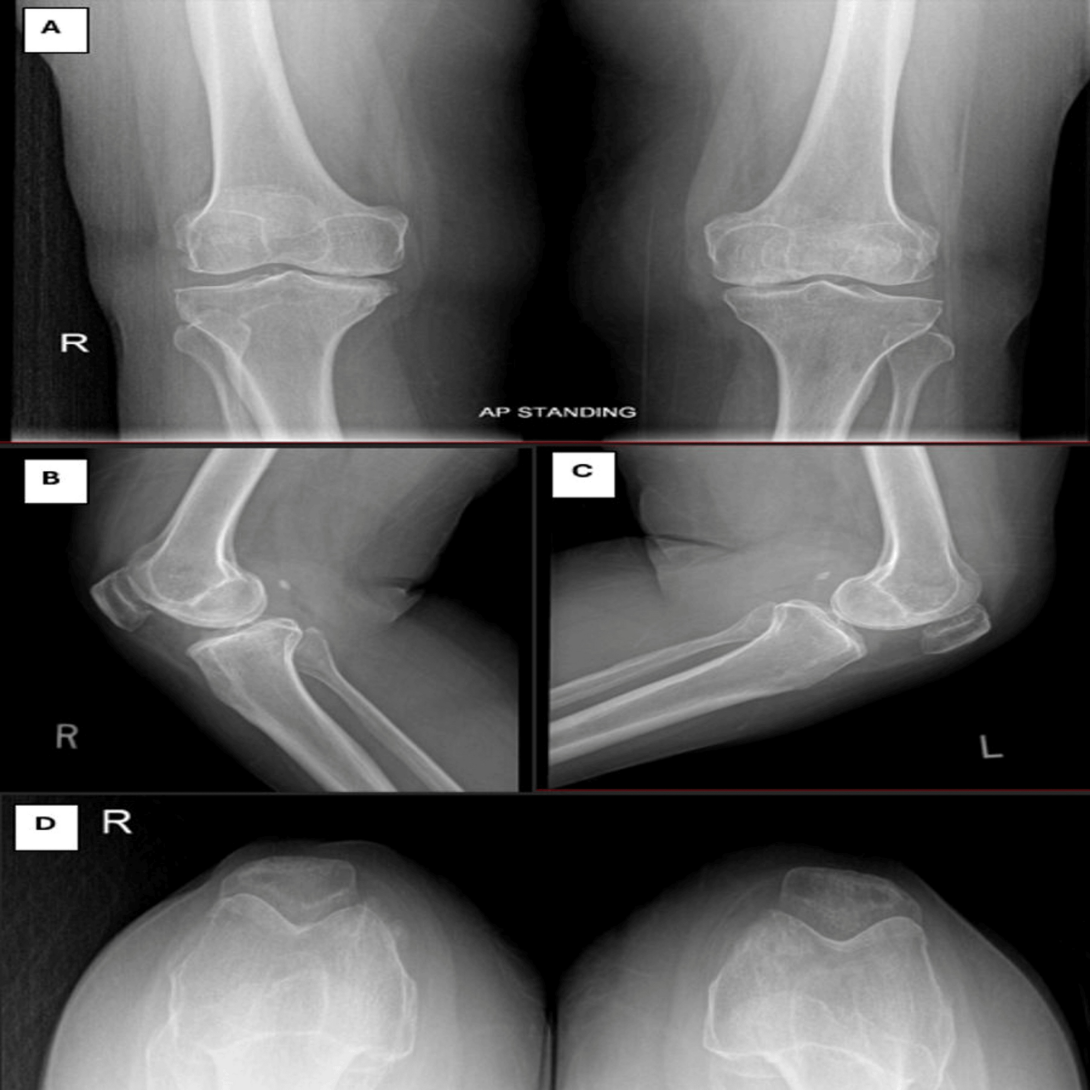
Bilateral knee radiograph showing severe bilateral knee osteoarthritis (A: anteroposterior, B: right knee lateral, C: left knee lateral, D: skyline view)

**Figure 2:**
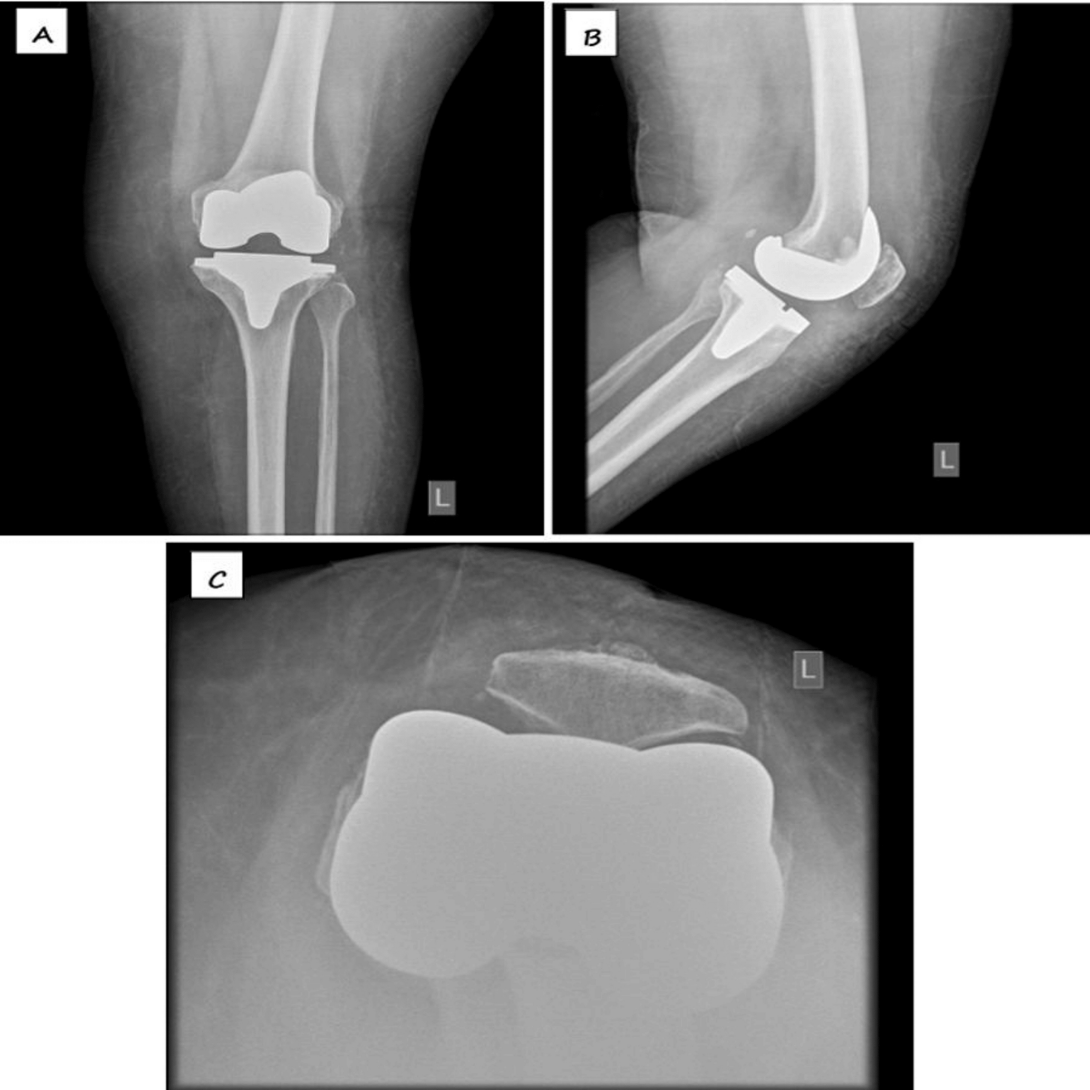
Left knee radiograph post left TKR showing well-aligned and fixed components with restoration of joint space (A: anteroposterior, B: lateral, C: skyline view) TKR: Total knee revision

A 62-year-old woman, with no known chronic medical illness, initially presented with bilateral knee OA on 23/10/2017 (Figure 1). Due to severe OA, she underwent bilateral total knee revision (TKR). The left TKR was performed seven years ago (Figure 2), followed by the right TKR 10 months later. Both procedures were done using the MPP approach.

**Figure 3:**
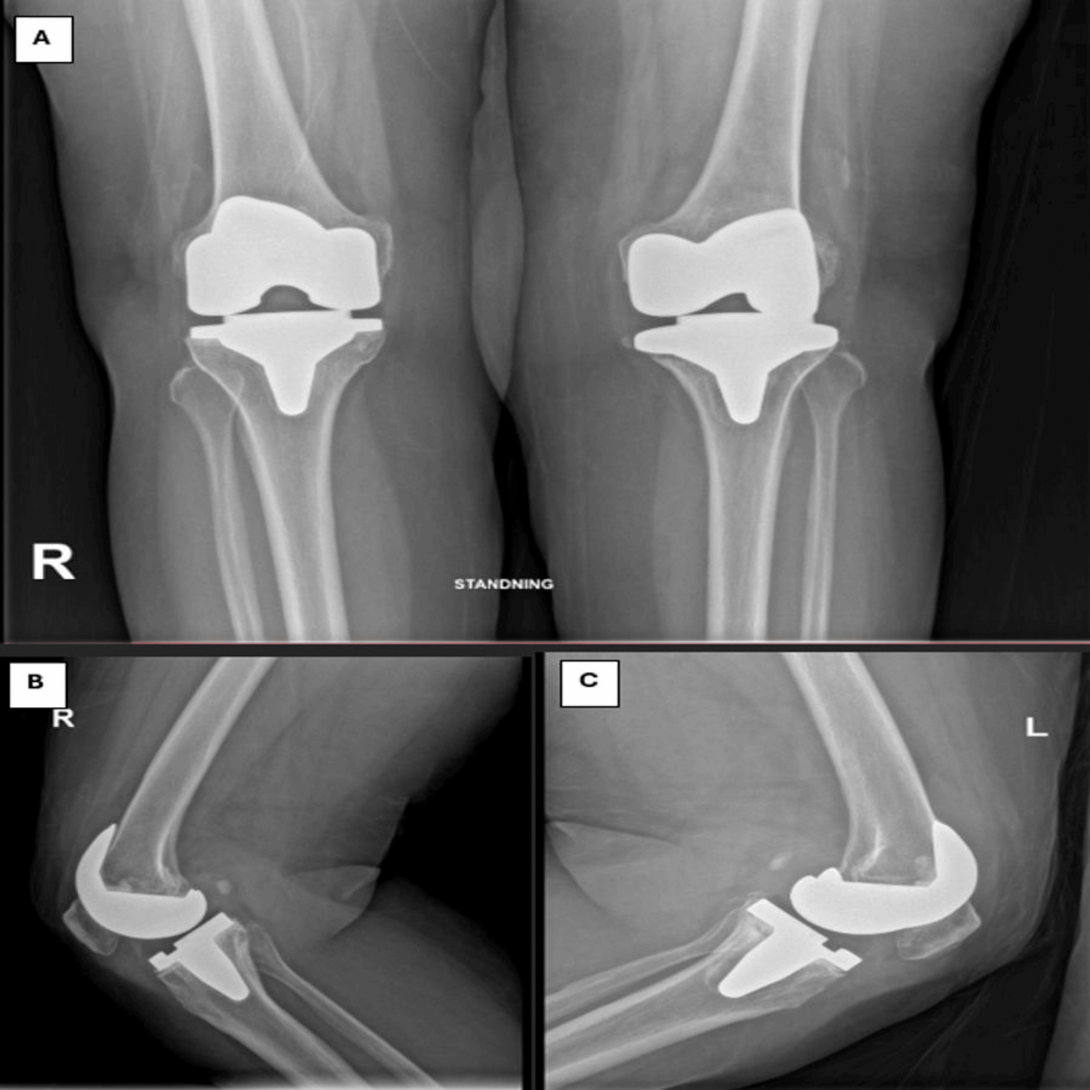
Knee radiograph post bilateral TKR showing right knee TKR post five years showing well-aligned and fixed components with adequate joint space, while the left knee TKR post six years demonstrating obvious signs of tibial component loosening, femoral component looks stable with no signs of loosening (A: anteroposterior, B: right knee, C: left knee) TKR: Total knee revision

Approximately three years after the left TKR, the patient began experiencing left knee pain. Serial radiographs revealed signs of tibial component loosening (Figure 3). All septic workup done showed that all results were normal indicating that it is aseptic loosening. Given the chronic nature of her symptoms and radiologic findings, she was scheduled for revision TKR of the left knee, which was performed on 25/9/2024.

After obtaining informed consent and explaining the risks and benefits of the procedure, the patient was positioned supine and underwent spinal/epidural anesthesia. A high thigh tourniquet was applied at 350 mmHg. A midline incision was made, extending to 4 cm above and 4 cm below the patella. The MV approach was utilized to provide surgical exposure. To enhance visualization, the vastus fascia was split, and the lateral patellofemoral ligament was released. Additionally, the suprapatellar and infrapatellar fat pads were excised.

Adequate exposure of the joint was achieved. Intraoperatively, the femoral component was found to be well fixed, while the tibial component was loose and easily separated from the underlying cement. The cement itself was well fixed to the bone. There was no evidence of infection and no pus discharge, and a swab for culture and sensitivity was taken. The tibial component was removed manually, and the cement was extracted using an osteotome. Thorough irrigation was performed.

Tibial preparation was carried out by reaming the medullary canal up to size 12. After multiple trials, the appropriate sizing was confirmed, and ligament balance was achieved. Final implants were inserted using the Knee System: tibial baseplate size 2, stem size 60 × 12, augment size 4, and polyethylene insert size 3 × 16. The pre-existing femoral component was retained (size 5). A cocktail injection was administered, and the wound was closed in layers.

**Figure 4:**
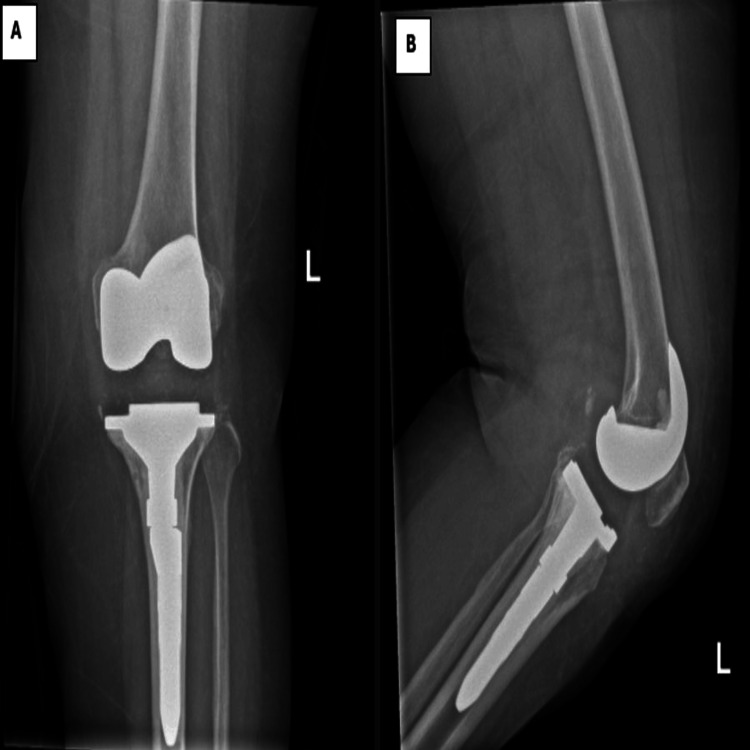
Left knee radiograph post left reversion TKR (rTKR) showing well-aligned and fixed components with restoration of joint space (A: anteroposterior, B: lateral) TKR: Total knee revision

The patient tolerated the procedure well and was transferred to the recovery room in good condition. Postoperatively, she began active ankle flexion and extension exercises on the same day. She received antibiotic prophylaxis for 48 hours and was allowed full weight-bearing from postoperative day one. A postoperative X-ray confirmed good alignment and preservation of the joint line (Figure 4).

**Figure 5:**
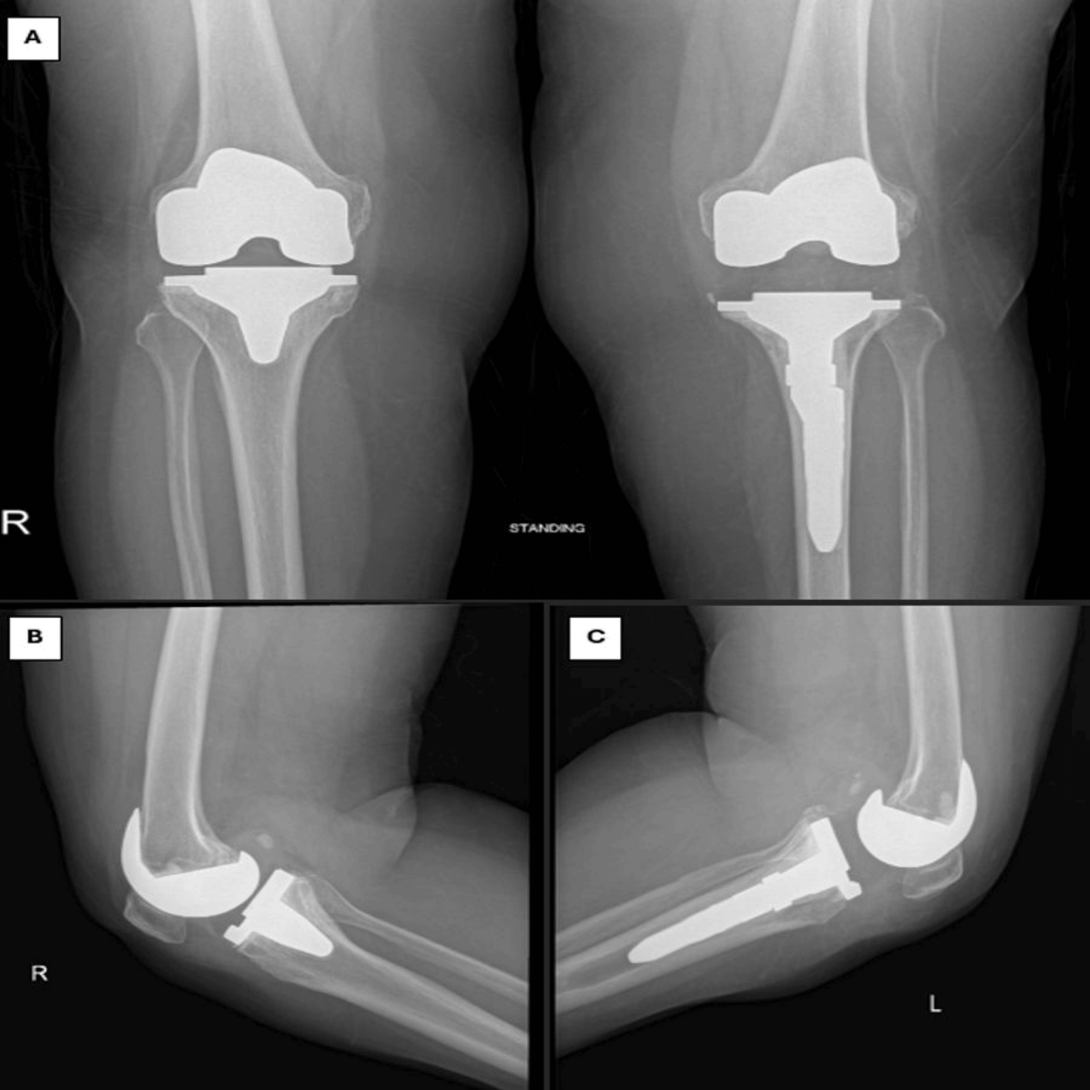
Bilateral knee radiograph three months post left reversion TKR (rTKR) and five years post primary right TKR showing well-aligned and fixed components with restoration of joint space of both sides (A: anteroposterior, B: right knee lateral, C: left side lateral). TKR: Total knee revision

The patient was discharged home on postoperative day three in good and stable condition, with a range of motion (ROM) of 0-90°. Wound culture and sensitivity swab were done, which showed no growth. During a routine clinic follow-up, the patient did not report any new complaints, she was compliant with physiotherapy and the wound remained dry and clean. Her ROM was 0-100°. X-rays showed proper alignment of the prosthetic components. Upon the last clinic follow-up (three months post op), the patient did not report any complaints and was actively demonstrating good ROM to 0-105°. The last X-rays (Figure 5) show good alignment of the prosthetic components. The patient planned to continue home exercises and the next outpatient follow-up was scheduled in three months (Figure 5).

## Discussion

This case report highlights the successful application of the MV approach in RTKA for aseptic tibial component loosening. While the MPP approach remains the conventional technique in revision settings due to the broad exposure it provides, our decision to utilize the MV approach in this case was based on growing evidence supporting its advantages in select revision scenarios, particularly where full extensor mechanism preservation and rapid postoperative recovery are priorities.

The patient, a 62-year-old woman with no significant comorbidities, presented three years after her primary left TKA with progressive pain and radiographic signs of tibial component loosening. Intraoperatively, the femoral component was found to be well-fixed and retained, allowing the revision to focus solely on the tibial side. Importantly, the MV approach offered sufficient exposure for extraction of the loose implant, debridement, and reimplantation of the new tibial component using modular augmentation and a press-fit stem. One of the major strengths of the MV approach is that it avoids disruption of the quadriceps tendon and minimizes trauma to the extensor mechanism [13,14]. This was clearly evident in our case, where the patient began active ankle exercises on the day of surgery, initiated full weight-bearing on postoperative day one, and was discharged home in stable condition by postoperative day three. By the three-month follow-up, she had achieved a painless ROM of 0-100°, well-aligned components on radiographs, and no wound-related complications, indicators of a smooth and functional recovery trajectory.

These postoperative outcomes are consistent with previous studies on the MV approach in primary TKA. Previous studies showed improved early functional outcomes, reduced pain, and faster quadriceps recovery without compromising implant alignment in the MV group [13,15,16]. While such benefits are well-documented in primary TKA, their extension into revision cases, as demonstrated in our report, offers a valuable contribution to current literature [16]. Despite this, our ability to perform complete tibial implant extraction and achieve stable reimplantation through the MV approach supports its viability when the surgical field is predictable and limited to one component. We enhanced visualization by carefully splitting the vastus fascia, releasing the lateral patellofemoral ligament, and removing the infrapatellar and suprapatellar fat pads. These adjunct maneuvers, when combined with the natural advantages of the MV approach, allowed safe eversion of the patella and unimpeded access to the tibial plateau.

Studies analyzing failure following TKA have distinguished between early revisions (within two years post-surgery) and late revisions (beyond two years). Polyethylene wear and subsequent aseptic loosening are the most frequently reported causes of late revisions [17], whereas infection and joint instability are predominant in early failures [18]. Furthermore, the association between morbid obesity and suboptimal outcomes in primary TKA is well established, with a growing number of younger, comorbid, and obese patients undergoing these procedures. This population shows significantly higher rates of postoperative complications such as infection, wound dehiscence, genitourinary issues, and mortality [19].

Another key advantage in our case was the preservation of the joint line and achievement of proper ligament balance. Trialing with different stem lengths and augment sizes allowed us to fine-tune the tibial alignment without needing to disrupt surrounding structures excessively. Previous studies emphasized the biomechanical importance of using modular stems in RTKA to ensure appropriate load distribution and implant longevity.

Importantly, no signs of infection were noted intraoperatively or during follow-up, supporting the decision for single-stage revision without use of antibiotic-loaded spacers. Early mobilization, rapid discharge, and continued improvement in ROM at successive follow-ups further underline the success of our chosen approach.

Nonetheless, this case must be interpreted within its limitations. A single-patient report cannot fully establish the efficacy or superiority of one approach over another. Furthermore, the favorable exposure achieved here was aided by the isolated nature of the tibial loosening. More complex revision scenarios involving multiple components, severe bone loss, or soft tissue compromise may still necessitate more extensile approaches such as MPP or even tibial tubercle osteotomy.

## Conclusions

This case demonstrates the effective use of the MV approach in RTKA for isolated tibial component loosening. By preserving the extensor mechanism and minimizing soft tissue disruption, this technique supported early mobilization and functional recovery. Intraoperative findings confirmed a stable femoral component, allowing a focused revision of the tibial side with successful implant integration and proper alignment. While not universally applicable, the MV approach may serve as a viable alternative in select revision scenarios where exposure needs are moderate and component failure is limited. Future perspective includes a broader evaluation of the MV technique in revision settings to validate its outcomes against traditional approaches. Lessons from this report highlight the importance of individualized surgical planning, implant modularity, and careful soft tissue handling in optimizing patient recovery.
